# Perceptual similarity and the neural correlates of geometrical illusions in human brain structure

**DOI:** 10.1038/srep39968

**Published:** 2017-01-09

**Authors:** Vadim Axelrod, D. Samuel Schwarzkopf, Sharon Gilaie-Dotan, Geraint Rees

**Affiliations:** 1 Inserm U 1127, CNRS UMR 7225, Sorbonne Universités, UPMC Univ Paris 06 UMR S 1127, Institut du Cerveau et de la Moelle épinière, ICM, F-75013, Paris, France; 2 Institute of Cognitive Neuroscience, University College London, London, UK; 3 Experimental Psychology, University College London, London, UK; 4 Visual Science and Optometry, Bar Ilan University, Ramat Gan, Israel; 5 Wellcome Trust Centre for Neuroimaging, University College London, London, UK

## Abstract

Geometrical visual illusions are an intriguing phenomenon, in which subjective perception consistently misjudges the objective, physical properties of the visual stimulus. Prominent theoretical proposals have been advanced attempting to find common mechanisms across illusions. But empirically testing the similarity between illusions has been notoriously difficult because illusions have very different visual appearances. Here we overcome this difficulty by capitalizing on the variability of the illusory magnitude across participants. Fifty-nine healthy volunteers participated in the study that included measurement of individual illusion magnitude and structural MRI scanning. We tested the Muller-Lyer, Ebbinghaus, Ponzo, and vertical-horizontal geometrical illusions as well as a non-geometrical, contrast illusion. We found some degree of similarity in behavioral judgments of all tested geometrical illusions, but not between geometrical illusions and non-geometrical, contrast illusion. The highest similarity was found between Ebbinghaus and Muller-Lyer geometrical illusions. Furthermore, the magnitude of all geometrical illusions, and particularly the Ebbinghaus and Muller-Lyer illusions, correlated with local gray matter density in the parahippocampal cortex, but not in other brain areas. Our findings suggest that visuospatial integration and scene construction processes might partly mediate individual differences in geometric illusory perception. Overall, these findings contribute to a better understanding of the mechanisms behind geometrical illusions.

Geometrical visual illusions have fascinated psychologists and laymen for centuries^1^. Numerous proposals explaining the reasons for illusory effects have been advanced, but no clear consensus has been reached^2^. Empirical research on illusions has been confined mostly to the exploration of each illusion separately, by manipulating their configurations (e.g., changing the shape of arrows in the Muller-Lyer illusion^3^). In a quest for unifying mechanisms of illusions, prominent theoretical proposals have been made^4^,^5^,^6^,^7^. However, empirically, only a few studies have examined the similarity between illusions^8^, most likely because there is no simple way to compare illusions that are visually very different. Notably, comparing illusions is an essential step forward because finding a similarity between specific illusions would imply that these illusions are likely to share common mechanisms^8^. In addition to behavioral investigation, neuroimaging methods can also help to better understand the mechanisms of geometrical illusions^1^. Several studies in the past explored the neurobiological basis of illusions with a functional MRI (fMRI) or event-related potential (ERP) design^9^,^10^,^11^,^12^, where a stimulus with an illusory effect was contrasted with a stimulus without an illusory effect (i.e., baseline). However, because visual (i.e., physical) stimulation between a condition with illusion and baseline condition differ, we cannot be sure the difference found at the neural level is related only to illusory processing. Furthermore, a condition with an illusory effect might also have a higher level of attention and/or arousal than a baseline condition. Thus, the use of this straightforward design (i.e., illusory vs. non-illusory conditions) cannot guarantee that illusory effects are specifically elucidated. In the present study, we examined the similarity between geometrical illusions and explored their potential neural mechanisms while we address the aforementioned limitations.

Fifty-nine healthy volunteers participated in our study, which included behavioral testing and structural MRI scanning. We tested four geometrical illusions: Muller-Lyer, Ebbinghaus, Ponzo, and vertical-horizontal illusions as well as a contrast illusion, as a non-geometrical control condition (Fig. 1). Using behavioral testing for each participant and illusion we established a participant’s individual illusion magnitude. To evaluate similarity among illusions, we correlated the magnitude of the illusions and explored shared variance among illusions by capitalizing on the variability of the magnitude of the illusion across participants^13^. The major benefit of this approach is that it permits us to compare very visually dissimilar illusions. In addition, to examine the potential neural mechanisms underlying geometrical illusions we correlated between the behavioral illusion magnitude and the gray matter density in the brain (voxel-based morphometry analysis^14^). The benefit of this approach is that in contrast to most previously used fMRI/ERP designs in our approach only the actual illusion effect is compared across participants, thus permitting us to elucidate the neural underpinnings of the illusions in a more specific way.

The main hypothesis that we tested in our study was that the geometrical illusions share a common mechanism. More specifically, while the research of geometrical illusions of recent years has mostly focused on the role of low-level visual processing (i.e., processing in the early visual cortex; for review, see ref. 15), here we asked whether more high-level, visuospatial mechanisms might also play a role in the misjudgment of geometrical illusions. That is, according to prominent misapplied size-constancy scaling theory^16^,^17^, the illusory effect for illusions like Muller-Lyer, Ponzo, and vertical-horizontal might be related to erroneously perceiving the lines of different distance, while the visual system implicitly transforms the 2-D image to a 3-D scene^3^,^18^. Furthermore, geometrical illusions like Muller-Lyer, Ebbinghaus, and Ponzo can be conceptualized as complex scenes, consisting of target elements (i.e., elements that are compared) and spatial inducer elements (i.e., elements that influence the illusory percept). Accordingly, the illusory percept in these illusions might be a result of inconsistency in visuospatial context integration^19^,^20^,^21^,^22^,^23^ and the construction of a spatial scene^24^,^25^. At the neural level, we focused primarily on the parahippocampal cortex (PHC) and the hippocampus – regions that have been extensively implicated in visuospatial, scene-integration and spatial processing in general^24^,^26^,^27^,^28^,^29^,^30^. Thus finding a correlation between gray matter density in the PHC/hippocampus and magnitude of geometrical illusion will support the view that visuospatial integration and scene construction process might play a role in perception of geometrical illusions. In addition, to investigate the regional specificity of the PHC/hippocampus, we examined a number of control regions that have been implicated in scene and visual processing in the temporal, occipital, and parietal lobes, including the retrosplenial cortex (RSC) and the transverse occipital sulcus (TOS) scene-selective regions.

## Results

### Behavioral results

**Figure 1:**
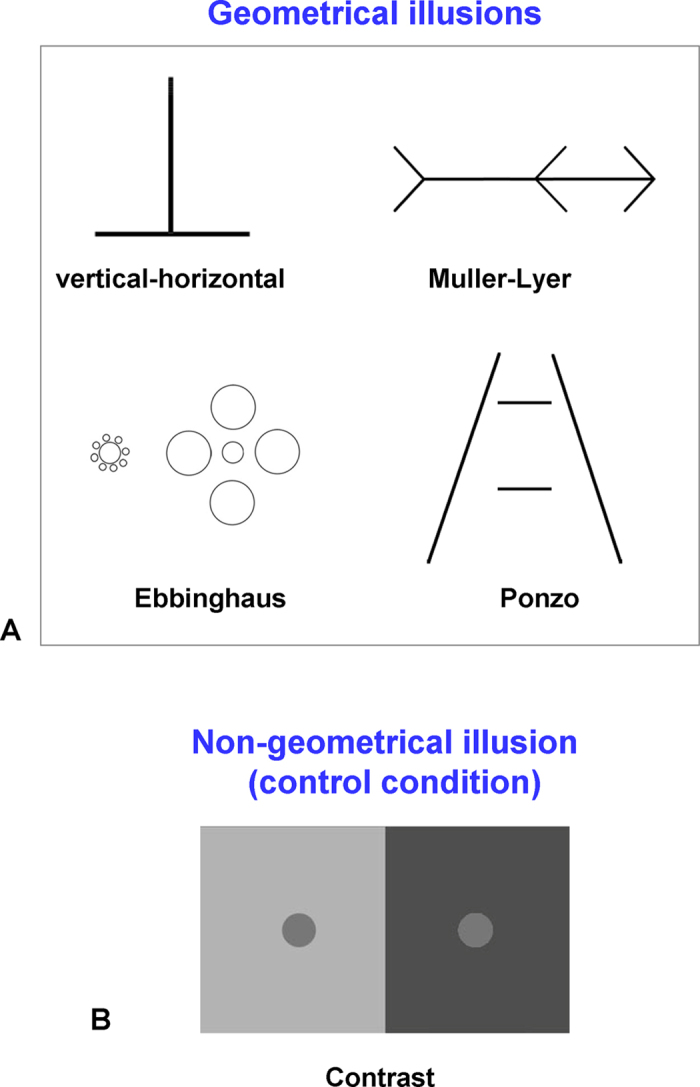
Illusion stimuli used in the experiment. (**A**) Four geometrical illusions: vertical-horizontal, Muller-Lyer, Ebbinghaus, and Ponzo illusions. To measure the illusory effect, participants were asked to manually adjust the correspondent segments so two corresponding parts of a figure would appear to them as perceptually equal. In the vertical-horizontal illusion, participants adjusted the vertical bar; in the Muller-Lyer illusion, participants adjusted the position of the central arrow; in the Ebbinghaus illusion, participants adjusted the size of the right central circle; and in the Ponzo illusion, participants adjusted the length of the top horizontal bar. Note that in the stimuli shown in the figure, the corresponding segments are equivalent (e.g., the left and right parts of the line in the Muller-Lyer illusion are equal). (**B**) Non-geometrical control contrast illusion. Participants were asked to adjust the brightness of the right circle, so it would have the same brightness as the left one. In the stimulus shown in the figure, the two circles are the same brightness.

**Figure 2:**
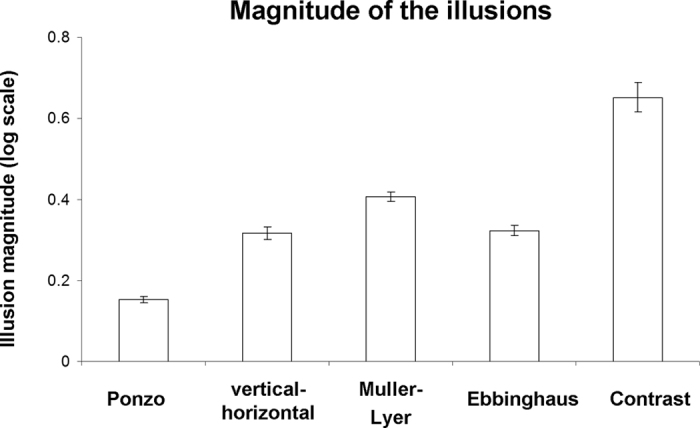
Illusion magnitudes (behavioral results). The illusory magnitude values reflect the binary logarithm (base 2) of the ratio between perceptual estimation and the real stimulus dimension (for geometrical illusions) or brightness (for contrast illusion). Values above zero reflect an illusory effect. Note, that illusory effects were found for all the illusions. Error bars denote standard error of mean. Individual illusory magnitude values can be found in Fig. 4.

For each of the four geometrical illusions (Fig. 1A) and the contrast illusion (Fig. 1B) participants made a manual adjustment until the two corresponding parts of the illusions were perceived as equal (for geometrical illusions) or of the same brightness (for the contrast illusion; see Methods for details). First, we validated that our realization of the illusions indeed resulted in an illusory percept. To be able to compare the results across geometrical illusions, for each illusion we computed the illusion magnitude, which was a log transformed ratio of subjective perceptual estimation vs. objective, real stimulus length or brightness (see Methods and refs 22,31). According to this scale, values above zero indicate an illusory effect. Average magnitudes for each illusion are shown in Fig. 2. We found illusory effects in all the illusions across participants: Ponzo (mean: 0.15, MSE: 0.007), vertical-horizontal (mean: 0.32, MSE: 0.01), Muller-Lyer (mean: 0.41, MSE: 0.012), Ebbinghaus (mean: 0.32, MSE: 0.013) and contrast (mean: 0.65, MSE: 0.03). Overall, implementation of the illusions was successful while an illusory effect was found in every single participant for the geometrical illusions and for each participant except one for the contrast illusion.

**Figure 3:**
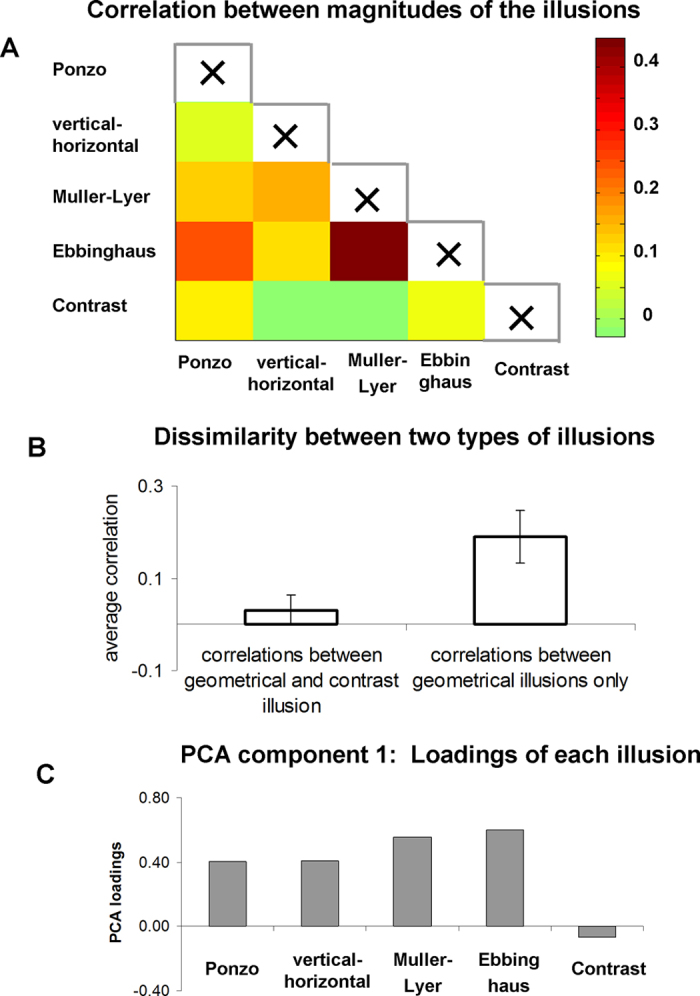
Similarity between illusions (behavioral results). (**A**) Correlations between magnitudes of the illusions across participants (Spearman correlation). Note that 1) the correlations between geometrical illusions was on average higher than correlations between geometrical illusions and contrast illusion; and 2) the strongest correlation was found between the Muller-Lyer and Ebbinghaus illusion. The scatter-plots of these correlations are shown in Fig. 4. (**B**) Comparison of average correlations of magnitudes of the illusions among the geometrical illusions (right), and between geometrical and contrast illusion (left). Note a clear difference between the two groups. Error bars denote standard error of mean. (**C**) Loadings of each illusion for the first PCA component that explained 36% of the total variance. Note a clear dissociation between geometrical and contrast illusions. The sign of the PCA loadings is arbitrary.

**Figure 4:**
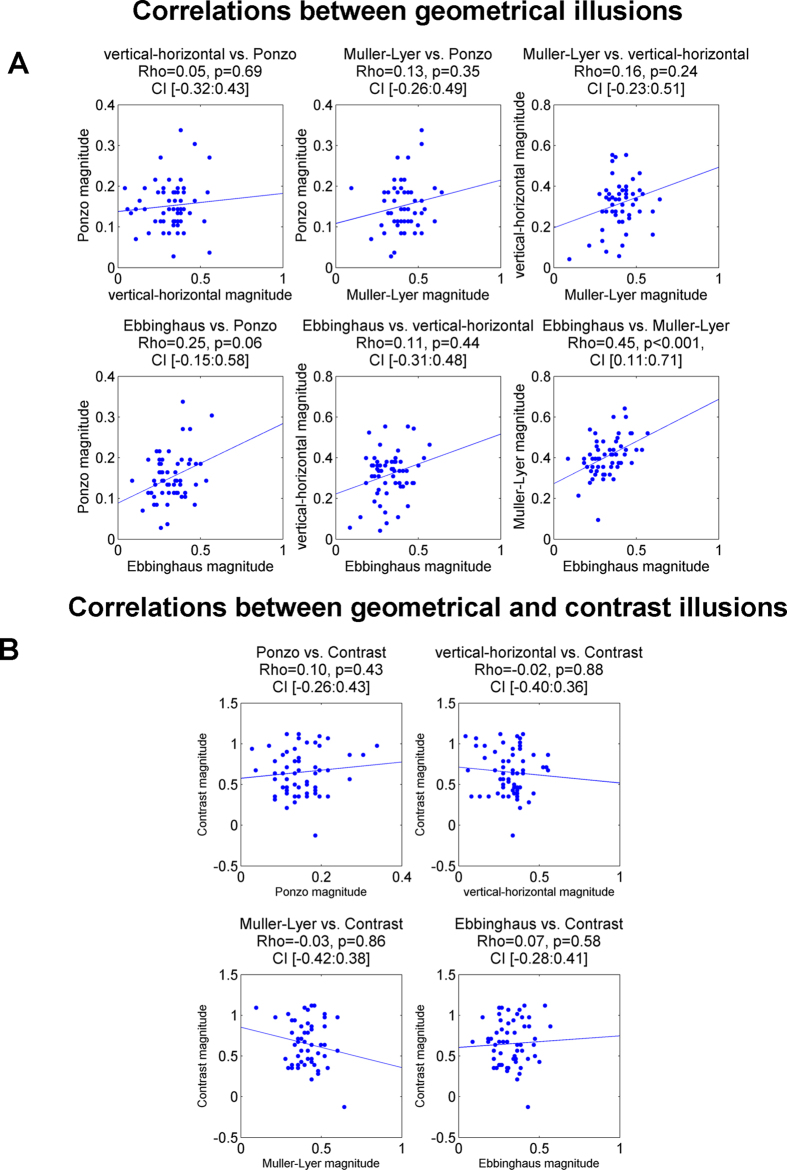
Scatter plots of correlations for all illusion pairs. (**A**) Correlation between illusory effects of geometrical illusions. Note the significant correlation between the Muller-Lyer and Ebbinghaus illusions. (**B**) Correlations between the illusory effects of the geometrical and the contrast illusions. The dots denote individual magnitude for corresponding illusion. The tendency lines were obtained as an ordinary least squares (OLS) fit. Statistical results are Rho of Spearman correlation, permutation p-values and 99.5% confidence interval using permutation analysis.

To examine the similarity between illusions, we calculated pair-wise correlations between the magnitude of the illusory effects across participants. In all correlation analyses we used Spearman rank correlation, a measure which is more robust compared to Pearson correlation (see Methods). Qualitatively similar results were obtained using skipped correlation^32^,^33^ and Shepherd correlation^34^. The pair-wise correlations between illusions are shown in Fig. 3A and Table 1. Scatter plots are presented in Fig. 4. Visual inspection of the correlation matrix (Fig. 3A) reveals that correlations between geometrical illusions were higher on average than correlations between geometrical and contrast illusions (more red and orange in the former than in the latter case). Further, while all correlations between geometrical illusions were positive (Fig. 4A), the correlations between geometrical and contrast illusions were around zero (Fig. 4B). The dissimilarity between two types of the illusions is best appreciated when the correlations between geometrical illusions and correlations between geometrical and contrast illusion are averaged (Fig. 3B). Because individual observations were not independent, we did not conduct statistical inference. To further explore the dissociation between geometrical and contrast illusion, we conducted Principal Component Analysis (PCA) for the illusory magnitudes for all five illusions (PCA input data: 59 participants × 5 illusions; see Methods). The first principal component explained 36% of the variance (for the full results of PCA analysis, see Table 2). Remarkably, examination of the loadings (i.e., weights) of this first component (Fig. 3C) revealed a clear dissociation between two types of illusions: while the loadings of the geometrical illusions were large in one direction, the loading of the non-geometrical contrast illusion was weakly in the opposite direction (the direction of the loadings is arbitrary in PCA). Thus, the first component reflects some common factor that is specific for geometrical illusions. Taken together, our results demonstrate similarity between geometrical illusions and also their dissociation from non-geometrical contrast illusion.

In addition, we examined more closely specific correlations between illusions (Figs 3 and 4 and Table 1). We found highly significant correlations (after Bonferroni multiple comparison correction, n = 10) between the magnitude of the Ebbinghaus and Muller-Lyer illusions: Spearman Rho = 0.45, permutation p < 0.001, permutation 99.5% confidence interval [0.11:0.71]. The correlation between the magnitude of the Ponzo and Ebbinghaus illusions was relatively high, but it did not reach statistical significance (R = 0.25, p = 0.06, 99.5% confidence interval [−0.15:0.58]). No significant correlations were found between other illusions (see, Table 1).

### Neuroimaging results

To determine the neural loci where brain structure covaried with the magnitude of the geometrical illusions, participants underwent anatomical MRI scanning. We used voxel-based morphometry (VBM) analysis to examine the correlation between local gray matter density and behavioral illusion magnitude (see Methods). Given our predictions that the processing of the tested geometrical illusions is related to visuospatial and scene-integration processing, we focused primarily on the parahippocampal cortex (PHC) and the hippocampus, which are regions that have been previously implicated in this type of processing^24^,^26^,^27^,^28^,^29^,^30^.

**Figure 5:**
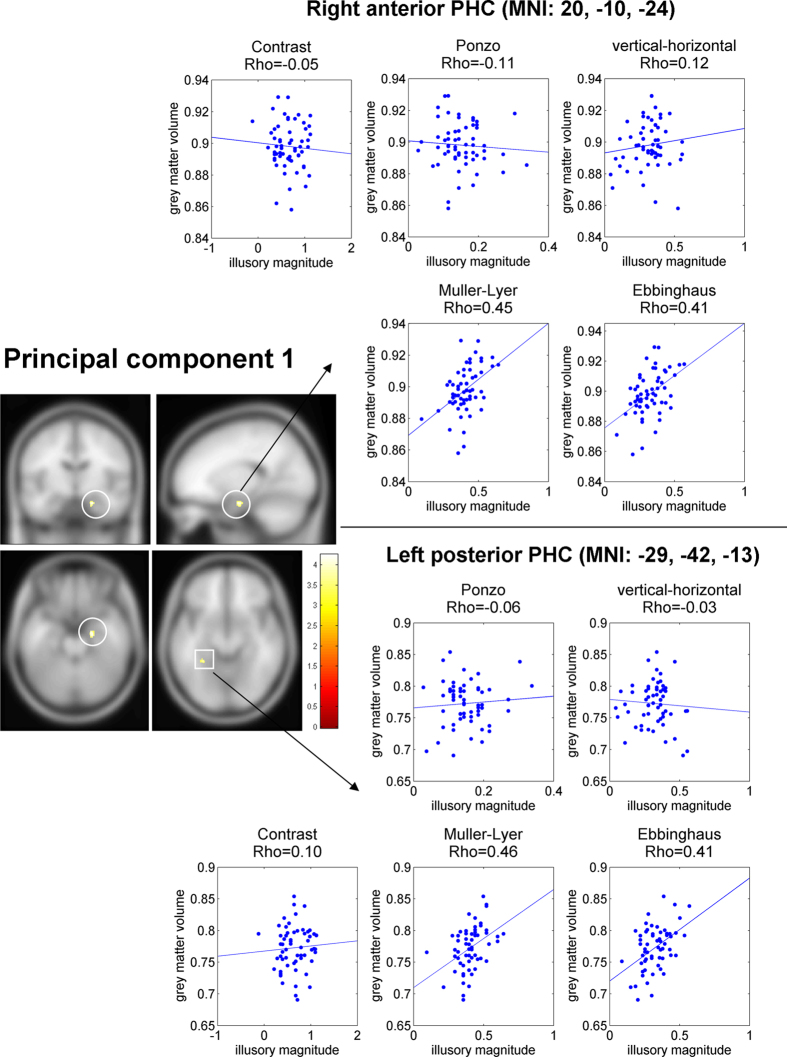
Result of VBM analysis for the first PCA component of the illusory magnitude and ROI correlation analyses for the significant clusters. Left: Two clusters in the right anterior parahippocampal cortex (PHC) and the left posterior PHC showed a significant correlation between local gray matter density and magnitude of first PCA component (p-value < 0.05, corrected). Right: scatter plots of correlation between gray matter density and illusory effect for the two clusters in the right anterior PHC and the left posterior PHC for four geometrical illusions. Each dot corresponds to an individual participant. Note, that because the behavioral values used for identifying the cluster (i.e., principal component of illusory magnitude) and behavioral values in the correlation (i.e., individual illusions magnitudes) were partially dependent, we did not conduct statistical inference of these correlations (i.e., no p-value or confidence interval was calculated). These correlations are presented only for visualization. Note the high correlation for the Ebbinghaus and Muller-Lyer illusions in both clusters. MNI coordinates of the clusters denote cluster center of mass.

Our behavioral analysis showed that the first principal component captured variability in illusion magnitude specific to geometric-illusions. Thus, we first asked whether local gray matter density of the PHC correlated with this component. In line with our prior hypothesis we found two significant clusters in the PHC (Fig. 5, left) where local gray matter density predicted the first principal component across participants. Specifically, the two clusters were in the right anterior PHC (MNI coordinates: 20, −9, −23; cluster size: 169 mm^3^, p-corrected = 0.009, Z = 3.89) and in the left posterior PHC (MNI: −32, −42, −14; cluster size: 81 mm^3^, p-corrected = 0.041, Z = 3.7). Next, to make a qualitative assessment of how specific these clusters were to each illusion, we: a) extracted gray matter density from these clusters, and b) correlated the gray matter density and the illusory effect for each of the four geometrical illusions. Note that because the behavioral values used for identifying the clusters (i.e., principal component of illusory magnitude) and behavioral values in the correlation (i.e., the magnitudes of the individual illusions) were partially dependent^35^, we did not assess the statistical significance of the correlations (i.e., we did not calculate p-values and confidence intervals). Our goal was to inspect and visualize the associations between gray matter density and the illusory effect for each of the four geometrical illusions. For a more detailed statistical explanation, see Methods. We found (Fig. 5, right), that in both clusters there was a high correlation for the Muller-Lyer and Ebbinghaus illusions. Interestingly, close to zero correlation was found for Ponzo and vertical-horizontal geometrical illusions. Thus, despite the fact that both Ponzo and vertical-horizontal illusions contributed to the first principal component (i.e., a common factor of geometrical illusions processing), other sources of variability in the Ponzo and vertical-horizontal illusions appeared more dominant. In addition, for both ROIs no correlation was found between gray matter density and contrast illusion (Fig. 5, right). Taken together, our central finding was that the neural structure of two loci in the PHC were related to processing of geometrical illusions.

**Figure 6:**
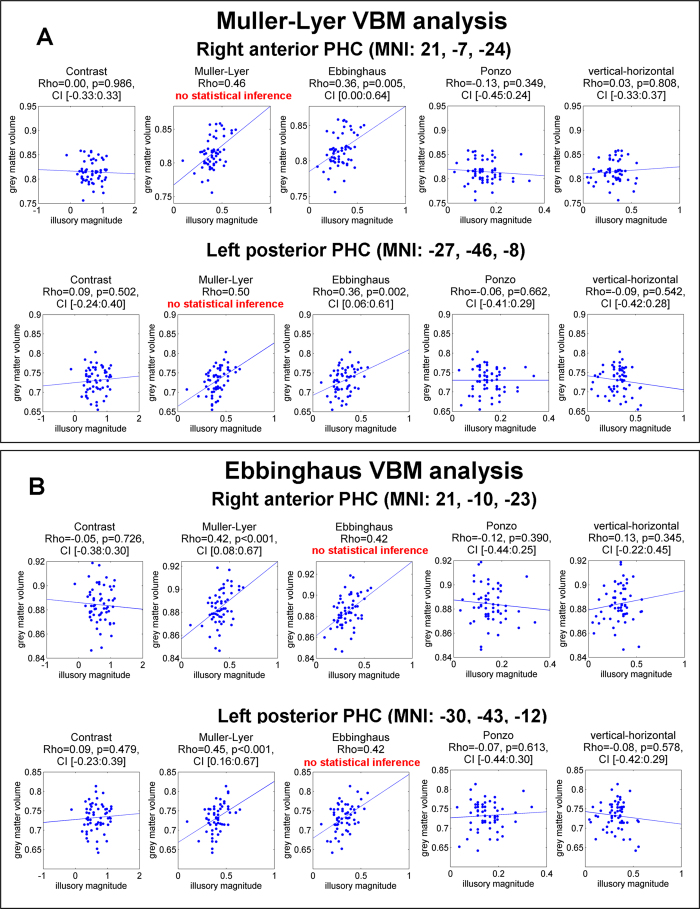
Scatter plots of ROI correlation between gray matter density and illusory magnitude for five illusions in the clusters that were identified using VBM analysis. (**A**) Correlations for clusters that were identified using Muller-Lyer illusion. No statistical inference was conducted for the Muller-Lyer illusion because the same data were used for identifying the cluster. The non-independent correlations are presented only for visualization. Note the high and statistically significant correlation for the Ebbinghaus illusion in both clusters. (**B**) Correlations for the clusters that were identified using Ebbinghaus illusion. No statistical inference was conducted for the Ebbinghaus illusion, because the same data were used for identifying the cluster. The non-independent correlations are presented only for visualization. Note the high and statistically significant correlation for the Muller-Lyer illusion in both clusters. Statistical results are Rho of Spearman correlation, permutation p-values and 99% confidence interval using permutation analysis.

To complement the VBM analysis with the first PCA component, we conducted VBM analysis for each of the illusions separately. To establish significance, we applied Bonferroni multiple-comparison correction for a number of geometrical illusions (p < 0.01, cluster level corrected). Analysis of the Muller-Lyer illusion revealed two clusters: left posterior PHC (MNI: −27, −46, −8; cluster size: 570 mm^3^, p-corrected = 0.002, Z = 4.07) and in the right anterior PHC (MNI: 21, −7, −24; cluster size: 435 mm^3^, p-corrected = 0.007, Z = 3.82). These two clusters were in close proximity with the clusters found using the first PCA component (right anterior PHC: the spatial overlap = 115 mm^3^, 68% of the first PCA component cluster and 26% of the Muller-Lyer cluster; left posterior PHC: the spatial overlap = 81 mm^3^, 100% of the first PCA component cluster and 14% of the Muller-Lyer cluster). Analysis of the Ebbinghaus illusion (p < 0.01, cluster level corrected) revealed a single significant cluster in the right anterior PHC (MNI: 21, −10, −23; cluster size: 216 mm^3^, p-corrected = 0.007, Z = 4.03). Using more relaxed threshold (p < 0.05, corrected) we also found a cluster in the left posterior PHC (MNI: −30, −42, −14; cluster size: 162 mm^3^, p-corrected = 0.027, Z = 4.15). Both these clusters were also in proximity with the clusters found using first PCA component (right anterior PHC: the spatial overlap = 149 mm^3^, 88% of the first PCA component cluster and 69% of the Ebbinghaus cluster; left posterior PHC: the spatial overlap = 81 mm^3^, 100% of the first PCA component cluster and 50% of the Ebbinghaus cluster). For clusters identified for Muller-Lyer and Ebbinghaus illusions we conducted ROI correlation analysis. Note, that to avoid circular analysis^35^, statistical significance of the correlations was established only for the clusters that were localized using another illusion. For example, for the clusters identified using the Muller-Lyer illusion, for the correlation between local gray matter and the Muller-Lyer illusion no statistical inference was conducted. The non-independent correlations are presented only for visualization. For a more detailed explanation, see Methods. In all identified clusters for both Muller-Lyer and Ebbinghaus we found high (around Rho = 0.4) and highly significant (p < 0.005) correlations (see, Fig. 6 for scatter plots and Table 3 for full results). The VBM analyses conducted for the Ponzo, vertical-horizontal and contrast illusions did not identify any significant clusters for the original threshold (p < 0.01, corrected) or for the more relaxed threshold (p < 0.05, corrected).

To examine regional specificity of the PHC with regard to a potential link to geometrical illusions, we tested in additional brain regions the correlation between local gray matter density and behavioral illusion magnitude. The regions were defined based on the coordinates previously reported in the literature (see Methods). First, given that our hypothesis linked the illusory effects to scene construction, we examined three well-established scene-selective regions^36^: the parahippocampal place area (PPA), the retrosplenial cortex (RSC), and the transverse occipital sulcus (TOS). Notably, the PPA is located at the posterior part of the parahippocampal cortex; therefore, some of the effects reported for the parahippocampal cortex were expected to be found for the PPA. Indeed, we revealed a significant cluster in the left PPA in the analysis of the first PCA component (MNI: −30, −42, −14; cluster size: 142 mm^3^, p-corrected = 0.004, Z = 3.66), Muller-Lyer illusion (MNI: −27, −46, −8; cluster size: 418 mm^3^, p-corrected <0.001, Z = 4.38) and Ebbinghaus illusion (MNI: −30, −42, −14; cluster size: 209 mm^3^, p-corrected = 0.003, Z = 4.12). The location of this cluster matched exactly the left PHC cluster that we found in the PHC analysis. For the Muller-Lyer illusion we found an additional cluster in the right PPA (MNI: 29, −37, −17; cluster size: 162 mm^3^, p-corrected = 0.007, Z = 3.73). This cluster did not reach significance in the analysis of the PHC/hippocampus, since the PHC/hippocampus volume was larger; consequently, the multiple comparison correction in the case of the PHC/hippocampus mask was more stringent. For the RSC and TOS regions, we did not indentify any significant clusters in the first PCA component or the separate illusion analyses (p < 0.05, corrected). In our second set of analyses, we examined the correlation in the lateral occipital (LO) and inferior intraparietal lobule (inferior IPL) regions because both of these regions exhibit higher connectivity with scene-selective regions^28^. In addition, since LO and inferior IPL were previously implicated in illusory brightness processing^37^, it was especially interesting to test whether the magnitude of the contrast illusion is associated with local gray matter in these regions No significant clusters for the first PCA component or for each illusion separately were found in these analyses (p < 0.05, corrected). Finally, we examined whether we could find a correlation between local gray matter density and behavioral illusion magnitude outside the predefined regions. To this extent, we conducted whole-brain analysis. Separate analyses of the first PCA component as well as of the five illusions revealed no significant clusters (p < 0.05, corrected).

## Discussion

In the present work we investigated potential mechanisms of geometrical illusions exploiting variability of the magnitude of the illusion across participants. We found clear similarity across participants between the behaviorally measured magnitudes of all tested geometrical illusions, but not between geometrical and a non-geometrical, contrast illusion. The highest similarity across participants was between the magnitude of Ebbinghaus and Muller-Lyer geometrical illusions. Furthermore, the illusory magnitude of all geometrical illusions, and particularly, Ebbinghaus and Muller-Lyer illusions correlated with gray matter density in parahippocampal cortex, supporting the view that visuospatial integration and a scene construction process might play a role in the generation of an illusory percept. Below we discuss how these findings contribute to an understanding of the mechanisms for geometrical illusions.

Geometrical illusions are a fascinating phenomenon. Surprisingly, despite decades of research, we still do not clearly understand the mechanisms underlying many illusions. Furthermore, it remains unknown whether different illusions are mediated by similar mechanisms. That is, while prominent theoretical proposals of illusion taxonomies have been advanced^4^,^5^,^6^,^7^, there have been few experiments that have compared between different visual illusions. The scarcity of empirical work addressing this question is probably not surprising, given that the illusions have very different visual appearances. Therefore, it is difficult to compare them directly. Here, we examined similarity between illusions by capitalizing on the variability of magnitude of the illusion across participants^13^,^38^. The major benefit of this approach is that it permits us to compare visually very dissimilar illusions. Using both principal component analysis and pair-wise correlation between illusions, we found that all tested geometrical illusions were similar to some degree (Fig. 3). At the same time, no similarity was found between geometrical and the non-geometrical contrast illusions. Notably, we designed our experiment so that the general structure of the tasks (i.e., comparison of two elements) and the adjustment procedure was similar and equivalent for all five illusions (both geometrical and non-geometrical illusions). Therefore a dissociation between geometrical illusions and the non-geometrical illusion was very unlikely to stem from unrelated cognitive factors^39^,^40^. Interestingly, an approach of comparing and correlating the strength of the illusions across participants was pioneered very long ago^8^. But since then it has been used only sporadically and only for comparison between two illusions^31^. The main motivation of this approach is that finding similarity between illusions implies that these illusions might share similar mechanisms^8^. If so, what are the possible common mechanisms of the Muller-Lyer, Ebbinghaus, Ponzo, and vertical-horizontal illusions?

In the present study we tested the hypothesis that processing of Muller-Lyer, Ebbinghaus, Ponzo, and vertical-horizontal illusions is associated with visuospatial context integration and scene construction. That is, all tested illusions consist of two or more elements that are perceived together as a coherent scene. The scene integration process might be particularly evident in the Muller-Lyer, Ebbinghaus and Ponzo illusions, where the surrounding figure elements (e.g., arrows in the Muller-Lyer) influence judgment of the target (e.g., the line length in the Muller-Lyer). In addition, it has also been repeatedly suggested that the illusory phenomenon might arise from an inappropriate interpretation of a 2-D image as a 3-D scene^3^,^5^,^18^. The most explored flavor of this idea has been the misapplied size-constancy scaling theory^16^,^17^ that proposed potential explanations for Muller-Lyer, Ponzo, and vertical-horizontal illusions. Given the well-established role of the parahippocampal cortex and hippocampus in visuospatial processing^27^ and scene construction^24^,^29^, we conjectured that finding a neural correlate of geometrical illusions in this region would support the hypothesis that visuospatial integration and scene construction processing might contribute to illusory percept. Remarkably, we found a reliable and significant correlation between local gray matter in the PHC and the first principal component of illusion magnitude – a component that had high coefficients for all geometrical illusions (Fig. 5). In addition, for both Ebbinghaus and Muller-Lyer illusions we found a high and significant correlation between local gray matter in the PHC and illusion magnitude (Fig. 6). The correlation was found only in the PHC, but not in scene-selective RSC and TOS, object-selective LO, or the inferior IPL. In addition, no significant clusters were found using whole-brain analysis. Critically, in contrast to previous studies, the method we used here permitted to delineate more specifically the neural correlates of the illusion effect. That is, previous studies used fMRI and ERP designs that contrasted two types of stimuli: with illusory percept vs. without illusory percept^9^,^10^,^11^. The limitation of previous designs is that when an illusory stimulus is contrasted with a stimulus without an illusion (i.e., a baseline), it is difficult to rule out the possibility that the difference between the two conditions is not related to unrelated (i.e., confounding) factors, such as higher arousal or selective attention in the illusory condition. The design we used was not based on a comparison versus baseline and therefore it permitted us to delineate the illusory effect in a more specific way. Taken together, our results provide support to the notion that visuospatial integration and scene construction process might contribute to generation of geometrical illusions effect.

We found that the first principal component of illusion magnitude, the component that had high coefficients for all geometrical illusions, significantly correlated with local gray matter volume in the PHC. Thus, all four geometrical illusions had some common source of variability that correlated with local gray matter volume in the PHC. This is consistent with all four geometrical illusions having some common neural mechanism related to the structure of the PHC. Having said that, given that the first principal component explained only part of the variance across illusions (36%), a substantial part of the neural correlates of this variability in magnitude of each illusion could be illusion-specific. Accordingly, it is quite conceivable that at the level of individual illusions, the magnitude of some illusions will not correlate with the local gray matter in the PHC. Indeed, we found that while the magnitude of the Ebbinghaus and the Muller-Lyer illusions had a high and significant correlation with local gray matter density in the PHC, no correlation was found for the Ponzo and vertical-horizontal illusions. This result was also in line with relatively lower loadings of the PCA first component for the Ponzo and the vertical-horizontal illusion (Fig. 3). Thus, while both the Ponzo and vertical-horizontal illusions had a component related to the mechanism in the PHC that was common across geometrical illusions, the relative weight of this component in these illusions was smaller. It is also possible that our experiment was not sensitive enough to reveal the visuospatial integration and scene construction in the Ponzo and vertical-horizontal illusions. Specifically, with regard to Ponzo − an illusion which is largely associated with 3D processing^17^ − a possible reason for our finding might be a relatively low illusion magnitude of this illusion (Fig. 2). As a result, the detection of any brain-behavior relationship would be more susceptible to unshared variance, such as measurement error. The magnitude of the Ponzo illusion we obtained was within the range of what is reported in the literature for this type of simple line stimuli^22^. The stimuli in our study were deliberately designed to be very simplistic to make the illusions more comparable. Thus, in the future, it would be interesting to specifically test the Ponzo illusion using a stimulus that generates a stronger illusion magnitude.

We have shown that the first principal component of illusion magnitude as well as the magnitude of Muller-Lyer and Ebbinghaus illusions correlated with gray matter in the PHC, a region that also includes the PPA scene-selective region. However, we found no significant correlation for any of the illusions or first principal component in the RSC and the TOS scene-selective regions. Thus, a potential mechanism for processing geometrical illusions was not associated with regions involved in scene-processing in general, but specifically with the PHC, including the PPA. In addition, we examined a correlation between magnitude of the illusions and local gray matter in the LO and the inferior IPL, but found no significant correlation in any two regions for the first principal component and any of the illusions, including the contrast illusion. It was particularly interesting to examine these two regions because they have been previously implicated in illusory brightness perception^37^. There are several possible reasons why, in contrast to the study by Perna and colleagues^37^, we did not find illusory brightness effects in the LO and the inferior IPL. First, Perna and colleagues examined the Cornsweet illusion, while here we used a contrast illusion, and the mechanisms supporting these two illusions might differ. Second, Perna and colleagues studied fMRI brain activations, while we examined anatomical local gray matter, and these are very different measures. That is, it is possible, that the specialization in brightness processing is not reflected in the neuroanatomy of these regions. Third, Perna and colleagues studied examined averaged activity, while our method is based on individual differences. Finally, the experimental designs employed in the two studies were very different. In particular, while Perna and colleagues compared the brain activity of different types of stimuli (e.g., edge stimuli, noise stimuli), in our study, the participants manually adjusted the stimulus in a behavioral experiment. The illusory scores were then correlated with local gray matter. Taken together, multiple factors could have contributed to differences between the results of the two studies.

It is important to emphasize that although we proposed and showed that a geometrical illusions phenomenon might be related to visuospatial integration and scene construction, we do not propose that this type of processing is the only process or factor that contributes to illusionary percept. That is, today, after many years of research, for the well-known illusions, such as the Ebbinghaus or Muller-Lyer, there are at least half a dozen potential explanations that are backed up by empirical results (for reviews, see refs 3 and 41). Critically, if we assume that only one of these explanations is correct, it is unclear how the current corpus of empirical evidence would be reconciled. For example, on one hand, different views emphasize the importance of the visual experience to experience illusory percept (e.g., natural statistics of visual stimuli^6^, culture-related experience^42^,^43^, and size-constancy scaling theory^16^). But on the other hand, it has been shown that individuals with bilateral congenital cataract are susceptible to geometrical illusions immediately after sight onset^44^. We think that a more likely scenario is that the illusions are mediated by several mechanisms (for similar ideas, see ref. 3), which might be realized in different cortical areas^15^. In the past decade, there has been a strong focus on the role of the early visual cortex in illusory perception. For example, activation of V1 reflects the perceived size of the illusory stimulus^45^,^46^,^47^,^48^,^49^ and the surface area of the V1 predicts the subjective experience of object size^31^,^50^,^51^. To this extent, our results complement these previous findings by showing that the regions associated with individual differences in illusory perception might be not only located in the low-level visual areas, but also in more high-level cortical regions.

We have shown that a larger density of local gray matter in the PHC correlated with a larger magnitude of geometrical illusions. How might the larger gray matter density in the PHC mechanistically contribute to higher illusory percept? Numerous studies in the past have linked higher gray matter density to more effective (or skilled) processing (for review, see ref. 13). Probably, the most relevant previous evidence is the case of London taxi drivers (i.e., people with extensive navigation experience), who had larger gray matter density in the posterior hippocampus compared to control participants^30^,^52^. In our case, a person with higher gray matter density in PHC might have higher level visuospatial integration and scene construction abilities. Such an individual might be less sensitive to the dimensions of individual elements, but rather to consider them as part of integrated scene. As a result, this might lead to a higher susceptibility to geometrical illusions. This tentative link will need to be tested in future research. Based on our findings, we would predict that patients with a lesion in the parahippocampal cortex would be less susceptible to geometrical visual illusions as their scene-integration mechanisms might be impaired. However, a focal lesion to the parahippocampal cortex is a relatively rare condition and, to the best of our knowledge, no study has yet examined geometrical illusions with such patients. With regard to spatial processing in general, one recent study found that patients with a lesion in the parahippocampal cortex were impaired at learning the spatial configuration of objects; but these patients were not impaired at learning the identity of objects^53^. So, this result is largely in line with the possible role of the parahippocampal cortex in scene construction and spatial integration.

Finally, as we already noted, the major benefit of the behavioral method that we used was the ability to evaluate similarity between visually very different illusions. That is, geometrical illusions have a very different appearance, and even if we hypothesize that two illusions are similar, there is usually no straightforward way to compare them. In addition to finding shared variance across all geometrical illusions (i.e., first principal component), we also found a high and significant correlation between the magnitude of illusory effects of the Ebbinghaus and Muller-Lyer illusions. Notably, the Ebbinghaus and Muller-Lyer illusions have very different visual configurations (e.g., central circles surrounded by more circles vs. a line with arrowheads). While these two illusions are two of the most explored illusions, we are unaware of any empirical attempt to examine their similarity. Here, using our correlation method, we demonstrated that these two illusions might potentially share similar mechanisms. As we have already discussed, the similarity between the Ebbinghaus and Muller-Lyer might be potentially explained by the mechanism of visuospatial integration and scene construction. Interestingly, an additional proposal suggests that the illusory effect of both the Muller-Lyer and Ebbinghaus illusions might be a result of incorrect comparison according to which the inducers are perceived as part of the target elements^5^,^54^,^55^,^56^. That is, the terminating left arrow of the Muller-Lyer illusion (Fig. 1A) might perceptually elongate the target line, and, consequently, the center of line is biased to the left side. Following the same logic, in the Ebbinghaus illusion (Fig. 1A), the surrounding circles of the left target might perceptually modulate the size of a target circle. The sources of such perceptual modulations in both illusions are not clear yet, but among other processes they might involve local interactions in early visual processing^49^,^51^. Critically, as we discussed previously, this poses no contradiction with our high-level visuospatial integration and scene construction view because the same illusion might be explained by several processes (i.e., mechanisms).

In conclusion, in the present study we found behavioral similarity between Muller-Lyer, Ebbinghaus, Ponzo, and vertical-horizontal geometrical illusions. We also showed that the illusory effect of all these illusions might be potentially explained by visuospatial integration and a scene construction process in the parahippocampal cortex. In particular, we report a high similarity between the Ebbinghaus and Muller-Lyer illusions.

## Methods

### Participants

Fifty-nine healthy volunteers with normal or corrected-to-normal vision participated in the study. Average age: 27 (MSE: 0.71) years; 30 females. All participants signed an informed consent form. The study was approved by the ethics committee of the Tel Aviv Sourasky Medical Center (Israel). All the methods were carried out in accordance with the these guidelines and regulations. Participants received either monetary payment or psychology course credit points for their participation in the experiment. All experimental procedures were performed in accordance with the guidelines provided by the ethics committee.

### Apparatus and software

Samsung notebook (NP350U2A), screen size 12.5”, 1366 × 768 resolution, and refresh rate 60 Hz, Microsoft Windows 7 Home Edition, Service Pack 1 operating system was used. The experiment was programmed as a Web application, running locally using an Apache 2.2 Web server (https://httpd.apache.org/) and Google Chrome browser. During the experiment, participants sat in a comfortable office chair; their distance from the monitor was 40 cm.

### Stimuli

Four geometrical illusions (i.e., vertical-horizontal, Ebbinghaus, Ponzo, and Muller-Lyer) and one non-geometrical illusion (i.e., contrast illusion) were used. Illusory stimuli are shown in Fig. 1. The stimuli were grayscale and were presented in the center of the screen. Illusory stimuli could be adjusted within some boundaries (i.e., a spectrum of possible adjustments). The boundaries were measured in a preliminary pilot experiment (different participants). In other words, the boundaries reflected the reasonable spectrum of variability across participants. While adjusting the stimuli participants were not aware of the boundaries (i.e., the adjustment limits). In addition, the boundaries were not reached by any of the participants in any of the illusions. The parameters of the stimuli were as the following. In the vertical-horizontal illusion the length of the horizontal line was 12° and the vertical line was adjustable, the minimal and maximal height was 12° and 17°. In the Ebbinghaus illusion, the radius of the inducers was 0.25° (left side) and 2° (right side). The radius of the left target circle was 0.9° and the right target circle was adjustable, and the minimal and maximal radius were 0.9° and 1.5°, respectively. The distance between the central circle and inducers for the left side was 0.1° and for the right side it varied between 0.5° (largest central circle) and 1° (smallest central circle). In the Muller-Lyer illusion, the total length of the line was 18°. The position of the central arrow was adjustable, and the maximal and minimal length of the left segment was 9° and 6.5°, respectively. The arrows had a length of 2.6° and a slope of 57°. In the Ponzo illusion, the length of the bottom horizontal line was 4.6°, and the top horizontal line was adjustable, with the minimal and maximal size 3.45° and 5.2°, respectively. The length of the surround lines was 18.7° and the slope of 21°. In the contrast illusion, the horizontal and vertical length of the whole figure was 20° and 12°, respectively. Left and right rectangles were of the same size and the circles were located at the centre of the rectangles; the radius of the central circle was 0.85°. The luminance of the surrounding part at the left and right side was 36.55 cd/m^2^ and 5.73 cd/m^2^; the luminance of the left central circle was 12.7 cd/m^2^ and the luminance of the right central circle was adjustable between 5.9 cd/m^2^ (most dark) and 15.9 cd/m^2^ (most light).

### Experimental procedure

Participants were shown an illusion figure with a short instruction text. Their task was using two buttons on the screen to adjust an element in the illusion figure until the two elements (fixed and adjusted one) appeared to them as perceptually equivalent. Adjustment was achieved by clicking buttons displayed on the screen with a computer mouse. In particular, for the vertical-horizontal illusion they had to change the length of the vertical bar so that it would appear to them as equal to the horizontal bar; for the Ebbinghaus illusion they had to resize the right circle so it would appear to them as equal in size to the left circle; for the Muller-Lyer illusion they had to move the central arrow to the center of the horizontal segment; for the Ponzo illusion they were asked to change the length of the top horizontal bar so it would appear to them as equal to the bottom horizontal bar; and for the contrast illusion they had to change the brightness (i.e., variations of gray) of the right circle so it would be the same as the left circle. Upon completing adjustment for a given illusion, participants clicked on the presented “OK” button that was constantly present on the screen. Then, they proceeded to the next illusion figure (see below). Participants were forbidden to approach their hands/fingers to the monitor or to use any auxiliary devices (e.g., ruler) while doing the experiment. The experimenter closely monitored the participants to ensure they performed the experiment based strictly only on their vision. Participants were also asked not to make calculations in their mind (e.g., mental rotation of the vertical-horizontal illusion), but to go with their intuitive perception (i.e., “gut feeling”). Each illusion was repeated several times (see below), while the starting configuration (e.g., arrow position of the Muller-Lyer illusion) varied between rounds. The starting configurations were uniformly sampled through the whole spectrum of possible configurations (i.e., the possible boundaries, explained above). By initiating the illusion adjustment from different starting positions, we minimized the influence of the starting position on the perceptual decision. Twenty-three participants completed four repetitions for each illusion and 36 participants completed five repetitions. The order of the illusions and the order of the starting configuration for each illusion were pseudo-randomized. No two examples of the same illusion appeared in direct succession. Before the main experimental session, participants performed a short training session, where each illusion appeared once. All participants confirmed they understood the instructions.

### MRI data acquisition

Structural MRI data (SPGR sequence) were collected for each participant using a 3 T GE MRI scanner (8-channel head coil) located at the Sourasky Ichilov Medical Center in Tel Aviv, Israel. Scanning resolution was 1 × 1 × 1 mm, providing full brain coverage, with TE = 3.52 ms, TR = 9.104 ms.

### Data Analysis

#### Behavioral data analysis

Data analysis was performed in MATLAB (R2009B version). The raw result values across several repetitions of the illusion were averaged. This resulted in a single raw value per participant/illusion. The magnitude of the illusory effect was calculated as a ratio between participant’s perceptual estimation and physically correct stimulus properties^22^,^31^. The ratio values were subsequently transformed using the binary logarithm (base 2) to correct for potential non-linearity of the data^57^. After transformation, values larger than 0 reflected an illusory effect^31^. Specifically, for each illusion the illusory magnitude (i.e., the ratio) was calculated as follows. For the Ponzo illusion, the length of the bottom horizontal bar divided by the length of the top horizontal bar. For the vertical-horizontal illusion the length of horizontal bar divided by the vertical bar. For the Muller-Lyer illusion the length of the right horizontal segment (until the arrow) divided by the left horizontal segment. For the Ebbinghaus illusion, the radius of the right center circle was divided by the radius of the left central circle. For Ponzo, the vertical-horizontal, and Muller-Lyer illusions the illusory magnitude was calculated for the length of the segments. Accordingly, for consistency, we also used the length measure (i.e., radius) for the Ebbinghaus illusion. We validated that when an area of the circle was used instead of radius, very similar results were obtained in all subsequent analyses.

Behavioral correlation analysis between the magnitude of the illusory effects was conducted using Spearman rank correlation. Spearman correlation is less sensitive to potential outliers than classical Pearson correlation^58^. In addition, Spearman correlation is a more preferable method because it can accommodate any monotonic relationship, whereas a Pearson correlation assumes a linear relationship. We also validated that qualitatively similar results were obtained when the robust skipped correlation^32^,^33^ and Shepherd correlation^34^ methods were used. We calculated significance p-values and confidence intervals using the bootstrap method^33^ (10,000 bootstrap sets^59^). To assess the significance of the correlations between illusions, Bonferroni multiple-comparison correction was applied based on the number of illusions. Confidence intervals were calculated for the percentiles by taking into account the Bonferroni multiple-comparison correction (i.e., 99.5% confidence interval for ten comparisons). Principal Component Analysis (PCA) for the magnitudes of the illusions (five illusions) was conducted using princomp MATLAB function (the input matrix: 59 participants × 5 illusions). Prior to submitting the data to PCA, for each illusion separately, the log-transformed magnitudes of the illusions were z-standardized (mean = 0, standard deviation = 1). The explained variance of each component was calculated as an eigenvalue of each component divided by the sum of eigenvalues. The scores of the first principal component (i.e., the representation of the input data in the principal component space) was used as a regressor in VBM analysis (see below).

#### Imaging data analysis

The structural data processing pipeline was the same as in our previous publications^60^,^61^,^62^. The data were analyzed using mainly SPM 8 (Wellcome Trust Centre for Neuroimaging, London, UK; http://www.fil.ion.ucl.ac.uk), except for the localization of significant clusters, which was performed using the “Non-Stationary Cluster Extent Correction for SPM” toolbox in SPM 5 (see below). Structural anatomical images were segmented to gray and white matter using a unified segmentation algorithm^63^. Then, an inter-subject registration of the gray matter images was performed using Diffeomorphic Anatomical Registration through the Exponentiated Lie Algebra (DARTEL) SPM toolbox^14^,^64^. The resultant gray matter images were smoothed using the Gaussian kernel (FWHM = 8 mm) and then transformed to the MNI coordinate system (using a transformation matrix of segmentation step). Multiple regression model was estimated for first principal component (covariate of interest: scores of first PCA component). In addition, for each of five illusions separate multiple regression models were estimated (covariate of interest: magnitude of the illusory effect). Covariates of no interest (i.e., influence that was regressed out) in all models were age and gender of the participants and global gray matter density. Given our predictions that the illusory processing of the tested geometrical illusions might be related to visuospatial and scene-integration processing, we focused primarily on the parahippocampal cortex and hippocampus^24^,^27^,^28^,^30^. To define this region, a single binary mask of the bilateral parahippocampal and hippocampus was constructed based on the aal masks of these regions^65^ (http://fmri.wfubmc.edu/software/PickAtlas). The models estimated for the main analysis (Fig. 5) were restricted to this binary mask. To establish statistical significance, in all analyses we applied cluster-level correction using the “Non-Stationary Cluster Extent Correction for SPM” toolbox^64^ (http://fmri.wfubmc.edu/cms/NS-General). We applied non-stationary correction, since it has been suggested that the use of standard cluster-based random field theory might be inappropriate because there is local variation in smoothness in structural images^66^. In the VBM analyses of individual illusions, localization of significant clusters was conducted using Bonferroni multiple comparison correction for the number of illusions (p < 0.01, corrected; 0.05/5 illusions). When no significant clusters were found at this corrected threshold, data were inspected using a more liberal threshold (p < 0.05, corrected). Every use of threshold p < 0.05, corrected is specified explicitly in the text. In accordance with recommendations^67^, in all analyses the primary threshold was p < 0.001.

In addition to the main focus of our study, the parahippocampal cortex/hippocampus, we conducted VBM analyses for additional regions. This permitted us to establish regional specificity of the results found in the PHC. The regions included scene-selective parahippocampal place area (PPA), retrosplenial cortex (RSC), and the transverse occipital sulcus (TOS), object-selective lateral occipital (LO) complex, and the inferior intraparietal lobule (inferior IPL). These regions were defined as a sphere with a radius of 8 mm, centered in coordinates previously reported in the literature. In particular, the coordinates (in MNI space) for the PPA and RSC were used from ref. 66 and were as follows: left PPA: −27,−46,−15; right PPA: 30, −44, −14; left RSC: −16, −64, 13, and right RSC: 20, −63, 17. The coordinates for the TOS were used from ref. 69 and were as follows: left TOS: −33, −80, 19; right TOS: 34, −77, 19. The coordinates for the LO and inferior IPL were used from ref. 37 and were as follows: left LO: −43, −82, 8; right LO: 43, −82,8; left inferior IPL: −31, −86, 23, and right inferior IPL: 31, −86, 23. Note, that similar coordinates for these regions were reported in many other previous studies (e.g., refs 70, 71, 72, 73). To search for neural correlates outside the a-priori regions, for each illusion we estimated a model without anatomical restrictions.

For the significant clusters found using VBM analysis we extracted gray matter density using the MarsBar region of interest toolbox for SPM^74^ and custom code^75^. The extraction of gray matter was performed for each participant, resulting in one data point per participant/per cluster. The extracted gray matter density was correlated with the behavioral illusory effect of each illusion. We took special precautions to avoid potential circular (“double-dipping”) analysis^35^. Our general strategy was as follows: For the clusters that were identified using the same or partly the same behavioral data (i.e., non-independent correlation analysis), no statistical inference was conducted (i.e., no p-values or confidence intervals). These analyses were used only for qualitative (i.e., visualization and inspection) assessment of whether there was an association between the two variables. For the clusters, which were identified using different behavioral data (i.e., independent correlation analysis), we calculated correlation Rho, p-values, and confidence intervals. In general, our analysis pipeline included two types of VBM analysis: a) analysis where the first principal component of illusion magnitude was used as a regressor, and b) a set of analyses where each illusion magnitude was used as a regressor. For the clusters identified using the first principal component (Fig. 5), no statistical inference was conducted for the correlation analysis between local gray matter and individual illusion magnitudes (i.e., the behavioral data was partially dependent). Despite this partial dependence, inspection of the association between two variables was still informative. That is, while relatively high correlation could be found for some illusions, no correlation was found for other illusions. For the clusters identified in the VBM analysis using the Muller-Lyer illusion, no statistical inference was conducted in correlation analysis between local gray matter and Muller-Lyer illusion magnitude (Fig. 6A). For the two clusters that were identified Muller-Lyer illusion, we conducted set of statistical correlation analyses using four remaining illusions (Fig. 6A). There is no circularity in this type of analysis because the identified cluster reflected strong correlation between local gray matter density (i.e., variable 1) and the behavioral scores of the Muller-Lyer illusion (i.e., variable 2). Then, gray matter volume in this cluster was correlated with the behavioral scores of the let’s say Ponzo illusion (i.e., variable 3). Because variables 2 and 3 are independent, the selection of a cluster based on the correlation between variables 1 and 2, does not introduce a bias for the correlation between variables 1 and 3. In the supplementary material, we provide a MATLAB simulation code that illustrates this idea. Finally, for the clusters identified using the Ebbinghaus illusion, no statistical inference was conducted in correlation analysis between the local gray matter and the Ebbinghaus illusion magnitude (Fig. 6B). The statistical analysis was conducted for the correlations between local gray matter and four remaining illusions (Fig. 6B; the same logic as for the Muller-Lyer illusion described previously). In all correlation analyses Bonferroni multiple comparison correction was applied to correct for the number of illusions (n = 5). Correlation analyses were conducted using the same methods as explained in the behavioral data analysis section.

## Additional Information

**How to cite this article**: Axelrod, V. *et al*. Perceptual similarity and the neural correlates of geometrical illusions in human brain structure. *Sci. Rep.* **7**, 39968; doi: 10.1038/srep39968 (2017).

**Publisher's note:** Springer Nature remains neutral with regard to jurisdictional claims in published maps and institutional affiliations.

## Figures and Tables

**Table 1:** Rho, p-values, and confidence intervals of Spearman correlations between illusions (behavioral results).

	Ponzo	Vertical-horizontal	Muller-Lyer	Ebbinghaus	Contrast
Ponzo	Rho = 1				
Vertical-horizontal	Rho = 0.05, p = 0.69, CI [−0.32:0.43]	Rho = 1			
Muller-Lyer	Rho = 0.13, p = 0.35, CI [−0.26:0.49]	Rho = 0.16, p = 0.24, CI [−0.23:0.51]	Rho = 1		
Ebbinghaus	Rho = 0.25, p = 0.06, CI [−0.15:0.58]	Rho = 0.11, p = 0.44, CI [−0.31:0.48]	**Rho** = **0.45, p < 0.001, CI [0.11:0.71]**	Rho = 1	
Contrast	Rho = 0.10, p = 0.43, CI [−0.26:0.43]	Rho = −0.02, p = 0.88, CI [−0.40:0.36]	Rho = −0.03, p = 0.86, CI [−0.42:0.38]	Rho = 0.07, p = 0.58, CI [−0.28:0.41]	Rho = 1

Confidence intervals (CI) and p-values were calculated using bootstrap analysis. Confidence intervals are 99.5%, to accommodate multiple comparison correction for 10 comparisons. Note the highly significant correlation p-value between the Ebbinghaus and Muller-Lyer illusions (marked with bold).

**Table 2:** PCA components’ loadings for each illusion.

	PCA 1 (36%)	PCA 2 (23%)	PCA 3 (17%)	PCA 4 (14%)	PCA 5 (10%)
Ponzo	0.40	0.48	0.37	−0.66	−0.20
vertical-horizontal	0.41	−0.25	−0.80	−0.35	−0.07
Muller-Lyer	0.55	−0.25	0.21	0.46	−0.61
Ebbinghaus	0.60	0.17	0.06	0.30	0.72
Contrast	−0.07	0.79	−0.42	0.37	−0.25

Variance explained by each component is given in brackets for each component.

**Table 3:** ROI correlation results for the two separate VBM analyses with Muller-Lyer and Ebbinghaus illusions, respectively.

Illusion used in VBM analysis	Identified cluster	Illusion used in ROI correlation analysis	Analysis results
Muller-Lyer	Right anterior PHC (MNI: 21, −7, −24)	Ebbinghaus	**Rho = 0.356, p = 0.005, CI [0.00:0.64]**
		Muller-Lyer	**Rho = 0.460**
		Ponzo	Rho = −0.127, p = 0.349, CI [−0.45:0.24]
		vertical-horizontal	Rho = 0.033, p = 0.808, CI [−0.33:0.37]
		contrast	Rho = 0.003, p = 0.986, CI [−0.33:0.33]
	Left posterior PHC (MNI: −27, −46, −8)	Ebbinghaus	**Rho = 0.360, p = 0.002, CI [0.06:0.61]**
		Muller-Lyer	**Rho = 0.503**
		Ponzo	Rho = −0.061, p = 0.662, CI [−0.41:0.29]
		vertical-horizontal	Rho = −0.085, p = 0.542, CI [−0.42:0.28]
		contrast	Rho = 0.085, p = 0.502, CI [−0.24:0.40]
Ebbinghaus	Right anterior PHC (MNI: 21, −10, −23)	Ebbinghaus	**Rho = 0.423**
		Muller-Lyer	**Rho = 0.417, p = 0.001, CI [0.08:0.67]**
		Ponzo	Rho = −0.118, p = 0.390, CI [−0.44:0.25]
		vertical-horizontal	Rho = 0.128, p = 0.345, CI [−0.22:0.45]
		contrast	Rho = −0.046, p = 0.726, CI [−0.38:0.30]
	Left posterior PHC (MNI: −30, −43, −12)	Ebbinghaus	**Rho = 0.421**
		Muller-Lyer	**Rho = 0.451, p < 0.001, CI [0.16:0.67]**
		Ponzo	Rho = −0.073, p = 0.613, CI [−0.44:0.30]
		vertical-horizontal	Rho = −0.078, p = 0.578, CI [−0.42:0.29]
		contrast	Rho = 0.086, p = 0.479, CI [−0.23:0.39]

The columns are: (1) the illusion used in VBM analysis for cluster identification; (2) the clusters identified in VBM analysis; (3) the illusion used for ROI correlation analysis and (4) the correlation analysis results. Note, that no statistical significance was determined when the same illusion was used for cluster identification and ROI analysis. Note the high correlations (marked with bold) for all clusters for Muller-Lyer and Ebbinghaus illusions. CI denotes 99% confidence intervals (accounting for multiple comparison correction, number illusions = 5).
